# Active versus passive reading: how to read scientific papers?

**DOI:** 10.1093/nsr/nwaa130

**Published:** 2020-06-19

**Authors:** Tung-Tien Sun

**Affiliations:** Departments of Cell Biology, Dermatology & Urology, New York University School of Medicine, USA

‘Any man who reads too much and uses his own brain too little falls into lazy habits of thinking.’ Albert Einstein (1879–1955)

‘Learning without thought is labor lost; thought without learning is perilous.’ Confucius (551–479 B.C.) The Confucian Analects, bk. 2:15

‘To repeat what others have said, requires education; to challenge it, requires brains.’ Mary Pettibone Poole, A Glass Eye at a Keyhole (1938)

## INTRODUCTION

‘How do you read a scientific paper?’ may at first seem like a superfluous question. Given how most biomedical research papers are structured,^1^ it might be natural for beginning (or even not-so-beginning) students to assume that one should first read the Title, then the Abstract, followed by the Introduction. Most might elect to skip the Methods section that customarily follows the Introduction (although many journals now place it towards the end of a paper), as it contains far too many technical details and is therefore boring. The Results section, which contains the meat of the paper, i.e., experimental data presented in the form of figures and tables, might receive the most attention, with the Discussion section that follows as a close second.

This kind of from-the-beginning-to-the-end and word-by-word reading is known as ‘passive reading’, which can be quite laborious and inefficient. In this paper, I will discuss the concept of ‘active reading,’ which I define here simply as *reading with questions in mind* and search for answers. In addition, you will ask yourself what experiments you would do next if you were the authors, and then compare your ideas with what was actually done in the paper.^2^ Reading this way fundamentally changes your mindset because the challenge keeps you intellectually engaged. You focus only on the parts of the paper that answer your questions, and glance through the rest; your reading therefore becomes highly selective. As a result, you can read faster and learn more. Although this paper will use biomedical research papers as examples, the same principles should apply to other scientific disciplines with minor modifications.

**Scheme 1.**
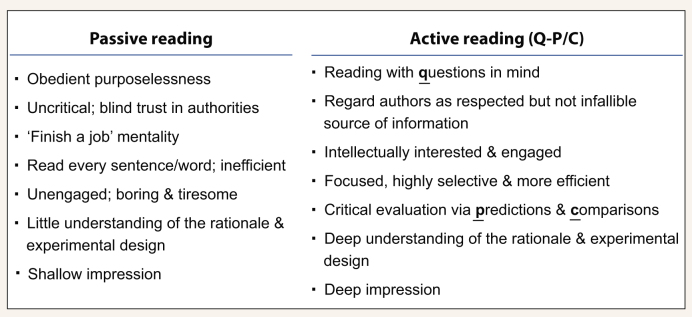
Distinguishing features of Passive vs. Active reading.

### Passive reading: ‘obedient purposelessness’

If you read a paper from-the-beginning-to-the-end, word-by-word, you are obediently following a path laid down by the author(s), who as a result, are de facto regarded by you as ‘authorities’ under this circumstance [1–3]. Reading a paper this way, without a particular expectation or purpose, is ‘passive reading,’ a practice similar to what Harvard educational psychologist William Perry (1913–1998) called ‘Obedient purposelessness’ [4]. It is inefficient and boring, because: First, if you set out with the intention to simply absorb whatever information that the author(s) have chosen to feed you, it is impossible, by definition, for you to be critical at the same time. Therefore, you lose the ability to assess the strengths and weaknesses of the paper. Second, because you are approaching the paper without expectations, reading through the paper will not likely generate any surprise or ‘Eureka’ moments and therefore becomes a chore. Third, while you might learn some facts and gain a general impression of the work, passively reading will not give you a deep understanding of the rationale and experimental strategies behind the work (Scheme 1).

### Active reading: reading with questions in mind

How can you improve the efficiency with which you do your reading? As an old saying goes, ‘you see only what you know,’ meaning you will be able to see an answer as such only if you were asking the question in the first place. Chinese scholar Huang Zongxi (黃宗羲, 1610–1695; [5,6]) once wrote: ‘If you read/do research with small questions in mind, you learn small things. If you do so with big questions in mind, you learn big things. *If you do so with no question in mind, you learn nothing*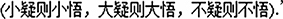.’ Reading with specific questions in mind puts you in the mindset of searching for little gold nuggets buried in a pile of sand. If you know what you are looking for, your eyesight is sharpened. You can coast along in a low-energy state while searching [7,8], and switch to a high-energy state (to read more thoroughly and intensively) only when you find what you are looking for. Reading in this way is highly selective, and it prolongs your attention span and is much more efficient than passive reading – which tires you out easily because you have to be on high alert the whole time. Moreover, the rush of excitement when you find the answers that you are seeking, particularly if they help solve a problem that you have or provide you with new ideas (the ‘Eureka’ moments) can be quite stimulating. Active reading is thus more productive and enjoyable than passive reading (for active vs. passive reading, see Scheme 1).

An obvious caveat to the above analogy of finding gold nuggets in a pile of sand is that you must first identify sand piles that are likely to be productive. Therefore, you should first practice active reading on papers that are published in highly selective, high caliber journals, at least while you are still learning the reading method.

## HOW TO DO IT?

### What kind of questions? A matter of ‘*Me-Me-Me*’

A requirement of active reading is for a reader to ‘know what he wants’, and to be able to ‘ask his own questions’ [7,9–12] – which is hard to do for many beginning students. But for most papers in the field of biomedical research, a novice active reader can ask the following four questions:

1. What is the question that the authors are trying to address, and why is it important?
2. If this were *my* thesis project, what kind of experimental approach can *I* take in tackling the problem?
3. What kind of data would *I* need to generate in order to support the conclusions of this paper?
4. How would this conclusion fit into *my* previous understanding of this subject?

**Scheme 2.**
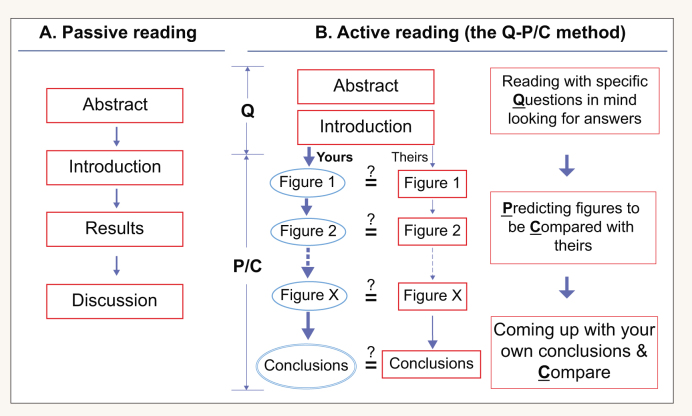
Reading scientific papers using the Q-P/C method (a form of active reading). One begins by reading the Abstract and Introduction with four specific questions in mind looking for answers. Based on this information and a brief literature search, one tries to design/predict the first experiment (Fig. 1; the left pathway) and compare it with the actual Fig. 1 that is published in the paper. This process of interrogation is then repeated for all other figures. One then tries to come up with her/his own conclusions based on the results, and compare them with what are described in the Discussion. The equal sign with a question mark denotes comparision. This method is based on Questioning-Predicting/Comparing, hence abbreviated as the Q-P/C method.

Notice that I have posted some of these questions from *MY* perspective. How would *I* tackle the problem, what data do *I* need, etc. Active reading is a self-centered process. Remember, I am reading because I want answers to *MY* questions!

### Reading the Abstract and Introduction: carefully and thoroughly

For papers in areas you are unfamiliar with, you are better off beginning with the Introduction, which may be more digestible than the Abstract which gives you an *overview* of the work, making it easier for you to navigate the paper. A well-constructed Abstract and Introduction almost always provide brief answers to all four questions raised above. If you encounter any words that you don’t understand, look them up immediately. Even a brief read of a Wikipedia page will greatly enhance your understanding and appreciation of the work. If you get lost while reading, backtrack a few sentences and try again.

### Answering the questions: usefulness of a brief literature search

After you finish reading the Abstract and Introduction, pause and digest what you have learned, and write down, *in your own words*, (preliminary) answers to the above four questions. Here it is highly advisable for you to *do a brief literature search on the subject and try to briefly read a couple of related, earlier papers.* This is beneficial because your ability to successfully predict figures as described below largely depends on the depth of your background knowledge and your familiarity with related studies. Having done your homework, you can then expand/improve your (‘final’) answers and move on to the predict-and-compare phase of the subsequent sections.

### Reading the Results: Predict-and-Compare

Armed with the in-depth answers to the four questions, ask yourself: ‘If this were my thesis problem, what would my first experiment, or set of experiments be, and what would my resulting Figure 1 look like?’ Draw upon the hints you got from your reading, you may decide the first experiment is to determine the tissue-specificity of a particular gene or to identify a group of specific protein binding partners.^3^ Based on this, you should draw on a piece of paper a schematic image of your hypothetic Figure 1, complete with various positive and negative controls and with as much detail as possible. Only then you go to the paper and compare your hypothetical figure with theirs. If your design is similar to theirs, you feel good about yourself. However, if you found that their design is better, you will have learned something valuable. Then, based on data in Figure 1, design what your second Figure 2 would look like. You continue this interactive process of Predicting-and-Comparing (P/C) until you have finished the whole Results section (Scheme 2).

You may have difficulty initially making any predictions, but with practice and an expanding knowledge base you should be able to improve.^3^ During this process, you will gradually gain confidence in your ability to learn about a new topic, and to design proper experiments in similar situations. As you do this, always try to draw your own conclusions based on the results, and compare your conclusions with theirs (Scheme 2).

Situations where you find yourself completely lost, with no idea what the next experiment(s) should be, actually offer the greatest opportunities for personal growth. Whenever this happens, resist the temptation to immediately read the paper to see what the authors did. Instead, do your best to come up with a solution yourself by looking up the relevant literature. Only after you have struggled through this process and made no significant headway should you read the paper. With anticipation and excitement, you turn the page and find out what the authors did. ‘Wow! That is brilliant!’ You might exclaim. They might have used a technique or a reagent unknown to you, to ingeniously unravel the mechanism of a reaction or a pathway. This strategy will now become a part of your toolbox, and you will never forget it. The more you have struggled to work through the problem on your own, the deeper is your impression of the newly acquired knowledge. There can be no gain without pain, after all.

The text in the Results section is primarily meant to explain the rationale for why the authors chose to conduct the experiments the way they did, and the logical links between them. Thus, if you can figure some of these out by yourself, you only need to glance through these parts of the text. It is only when you fail to predict a figure that you should read the relevant portion of the Results section to learn why the authors proceeded in a certain way.

An important consequence of this ‘Predicting-and-Comparing’ approach is that it enables you to gain a deep understanding of the strategies and rationale for the study, critically evaluate the authors’ experimental design and data, and judge whether their results support their conclusions.

### Reading the Discussion: comparing my conclusions with theirs

The Discussion is where the authors discuss why their data support the conclusions that they have drawn. If you agree with their interpretations, you can simply glance over this part of the discussion. The authors will likely also discuss how their data relate to preexisting knowledge, and the implications of this. For example, they may explain how their data lead to a new understanding of the problem, a new model that brings to light certain predictions, or novel practical applications. Because you have struggled through the experiments almost as the first author would have by reading the paper actively, you will particularly enjoy reading these discussions, which shows the rewards you can reap from this kind of project.

Since reading and writing may be regarded as two sides of the same coin [13,14], active reading can make you more aware of what you can do to help your fellow active readers find more quickly what they need. For instance, your abstract should be constructed so that it answers all four questions we raised. Each of your paragraphs should, where possible, start with a topic sentence outlining the contents of the paragraph [15]. You could also introduce your figure legends with an informative title phrased as a complete sentence, write the body of the legend with a minimal level of technical detail (these belong in the Methods section), and end with a concluding sentence highlighting the most salient findings of this figure.

## AN INTERACTIVE COURSE

Although some students will be able to achieve the transition from passive to active reading without much trouble by following the procedures outlined here, given the importance of the topic and possible difficulties some students may experience, a course on literature analysis for the graduate students in their beginning years^4^ dealing with this method, or some variation of it, could be helpful.

Since this course involves a significant amount of class participation and faculty-student interaction, it consists of about ten (biweekly) 1.5-hour sessions with a class size of no more than 16–20 students. A reading list of 10 papers on topics relevant to the graduate program could be put together, starting with several paradigm-shifting ‘classics’ so that the instructor can discuss the impact of these publications, and tell stories about some of the authors who are/were pioneers in the field.^5^ Moreover, these earlier papers are usually simpler to follow making it easier for the students to practice predicting the figures. In the first organizational session, the instructor can give a talk introducing Active Reading, describe what the students can expect from the course, and group students into teams of two for each paper. The students would then read the paper, and submit a report due the night before the class addressing the following:

- Preliminary and final answers to the four Questions
- Strengths and weaknesses of the paper in terms of the quality of the research question, the experimental design, the writing, and whether the data support the paper's conclusions
- Any thoughts s/he had while going through the paper

During the session, the instructor could ask the team assigned the paper being covered to start with a summary of the work (5–10 min), followed by discussing the strengths and weaknesses of the paper (5–10-min). All other teams then take turns presenting their critiques (5–10-min). The instructor should make clear early on that merely rehashing the main points made in the paper, or making superficial comments such as ‘I think the paper is great’ without further elaboration will not be sufficient here. During this process, the faculty could ask any presenter to explain the meaning of a keyword in the abstract/introduction, or how a particular experimental technique works, including its potential artifacts and limitations.^3^ At the end of the session, the instructor can present her/his own evaluation of the work, provide feedback on the discussion of the day, and allow the team in charge of the next paper to say a few words (5 min) to introduce the paper.^6^

## TWO UNIQUE FEATURES

The fact that we are dealing with the reading of only scientific papers allows us to design unusually detailed and practical guidelines that our students can follow. These guidelines have two unique features. Firstly, they allow the students to use the same set of four questions for all the papers. Secondly, they ask students to ‘predict’ what experiment should be done next and to compare their ideas with the authors. The combination of these two elements makes this method (the ‘Q-P/C method’) especially powerful. I will discuss these two features below.

### Questioning (Q)

Since active reading is widely accepted as an effective strategy to facilitate learning [7,16], many universities’ websites offer advice about how to do this (see, for example, [9,17–19]). They all emphasize the importance of surveying (a quick pre-reading to get an overall picture of the text) for the purpose of generating questions, before reading (plus recalling and reviewing; the ‘SQ3R’ or ‘KWL’ method [9–12]). The Achilles*'* heel of this method, however, lies in the questioning step, because beginning students often find it hard to come up with useful questions. Their recommended solutions range from ‘asking yourself what is the topic, what do you already know about it? why has the instructor assigned this reading at this point in the semester?’ [17]; to ‘turning paragraph headings into questions’ [18]; to ‘you are encouraged to glance ahead to the headings, charts, photographs, and so on to inform your questions’ [19]; and to ‘students should ask themselves what it is they want to get out of a reading assignment, then look around for those points’, and “students should ‘talk to themselves’ while reading, asking ‘is this the point I’m looking for?’ ” [4]. The diversity and vagueness of these recommendations illustrate how difficult it is to deal with this problem. In reading biomedical research papers, however, our students can sidestep this hurdle by asking the same set of (highly relevant) questions outlined earlier. In-depth answers to these questions give the students a foundation to proceed to the next part of the method, i.e., predicting-and-comparing.

### Predicting/Comparing (P/C)

The reading method described here makes extensive use of a ‘Predicting-and-Comparing’ strategy. Although students have been suggested to predict the text based on the headings, subheadings, figures, etc. [14,19], this practice has not been particularly effective. However, as part of the strategy presented here, ‘predicting’ means making an educated and highly specific guess as to how a figure should look, and comparing your ideas with the authors’. This method allows the students to critically evaluate the strengths and weaknesses of the experimental design. Moreover, for the students to interrogate the authors’ figures, it challenges the authors’ status as authorities [1–3]. Finally, by having the students involved in designing every experiment (Scheme 2), it is almost like they had completed the entire project – in the time it took for them to finish reading the paper instead of the months or years it had taken in real life.

## CONCLUDING REMARKS

Although relearning how to read papers may be a daunting proposition, active reading allows a student to gain a clearer understanding of a published study's experimental design and progression far better than passive reading ever could. Moreover, as you gain proficiency and as your knowledge base broadens over time, *you can streamline this process* and it will take you less and less time to read and analyze a paper. Eventually, you may be able to learn the essence of a new paper just by reading the abstract and looking through the figures, a process that may take you as little as 20–30 min, like most experienced investigators can do [20]. It will take time and effort to reach that goal, of course, but that's all the more reason why you should start as soon as you can.

## Footnotes

^1^
The principles described here are designed for reading papers that follow the conventional organization of having Abstract, Introduction, Results, and Discussion, but can also be applied with minor modifications for reading papers with other formats.

^2^
I have described this method in a lecture called ‘How to Read Scientific Papers and Generate Ideas’, one of the four lectures in my one-day Workshop on Scientific Methods that I gave at NYU School of Medicine and other institutes since early 2000s [21]. Another lecture on ‘How to Get Your Experiments to Work?’ was published in reference [2].

^3^
For you to be able to design the experiments properly, you must first possess a thorough understanding of the techniques that you use. You should be able to draw a schematic diagram showing exactly how these techniques work, and their limitations and potential artifacts. In the case of the PCR (polymerase chain polymerization), for example, you should know how the specificity, band patterns, and intensities will be affected by changes in Mg^2+^, and nucleotide and primer concentrations.

^4^
It may be beneficial to offer this course at an introductory level to first-year graduate students so they can learn how to use this method to read, but not to expect them to be able to use it proficiently in predicting figures, as it requires a significant familiarity with experimental techniques and the investigative process. A more advanced course can then be offered to second-year students with the expectation that they can improve their competence in predicting the figures and conclusions and derive enjoyment from such a process.

^5^
As Santiago Ramon y Cajal (1852–1934) said in *Advice for a Young Investigator* [22]: it is important ‘to trace the history of the problem. This is done…to render the tribute of justice to the scholars who have preceded us and opened the path of investigation for us.’ It is therefore important for you to know, initially at least for your own field of study, who the major players are and how their key findings have helped define the current paradigms in the field [23].

^6^
Since a major shift in the reading mentality is involved at the beginning of the course, students will experience various degrees of challenge depending on their attitude towards ‘authority’ [1–3]. It may, therefore, be helpful to arrange a few round-table discussions at suitable intervals during which students can share their thoughts and difficulties for the group's feedback and suggestions. The course can be graded based on the quality of the biweekly reports (depth of understanding), class participation, and a final essay (what has been learned and suggestions), taking into consideration the individual's learning trajectory. In addition, it may be useful to have 2–3 faculty members to either co-lead the class or to teach separate groups, and to work as a team to deal with various logistics and challenges [7], which this course aiming at changing students’ mentality of reading will surely face many.
